# Transmembrane protein 147 (TMEM147): another partner protein of *Haemonchus contortus* galectin on the goat peripheral blood mononuclear cells (PBMC)

**DOI:** 10.1186/s13071-016-1640-0

**Published:** 2016-06-23

**Authors:** Yan Li, Cheng Yuan, LiKun Wang, MingMin Lu, YuJian Wang, YuLing Wen, RuoFeng Yan, LiXin Xu, XiaoKai Song, XiangRui Li

**Affiliations:** College of Veterinary Medicine, Nanjing Agricultural University, Nanjing, 210095 People’s Republic of China

**Keywords:** Galectin, *Haemonchus contortus*, Goat PBMC, TMEM147, Member receptor

## Abstract

**Background:**

Recombinant galectins of male and female *Haemonchus contortus* (rHco-gal-m/f) have been recognized as significant regulators of the functions of goat peripheral blood mononuclear cells (PBMC). In previous research, transmembrane protein 63A (TMEM63A) was identified as a partner protein in the regulation associated with *H. contortus* infection. However, in the identification of binding partners for galectins of male and female *H. contortus* (Hco-gal-m/f) by yeast two-hybrid (YTH) screening, it was found that the transmembrane protein 147 (TMEM147) could also bind to Hco-gal-m/f. In this study, the functions of TMEM147 in the regulations of *H. contortus* galectin on the goat PBMC were investigated.

**Methods:**

To identify Hco-gal-m/f-interacting proteins, a yeast two-hybrid system to detect interactions was used. Co-immunoprecipitation and immunoblotting were used to validate the interaction between recombinant galectins of male *H. contortus* (rHco-gal-m) and candidate binding protein. The localization of TMEM147 in PBMC was explored by immunofluorescence in confocal imaging studies. Flow cytometry was used to determine the distribution of TMEM147 in T cells, B cells and monocytes in PBMC. The modulatory effects of rHco-gal-m and TMEM147 on cell proliferation, phagocytosis, nitric oxide production, migration, apoptosis and cytokine mRNA transcription were observed by co-incubation of rHco-gal-m and knockdown of the *tmem147* gene.

**Results:**

In this research, it was demonstrated that TMEM147 could bind to rHco-gal-m/f. Immunofluorescence assays showed that TMEM147 was localized to the cell membrane and within the cell membrane in goat PBMC. Flow cytometric analysis revealed that TMEM147 was expressed in all B cells and monocytes in goat PBMC. However, 3.8 % of T cells did not express this protein. Knockdown of the *tmem147* gene using RNA interference (RNAi) showed that the interaction of galectin with TMEM147 mainly mediated cell proliferation, cell apoptosis, transcription of interleukin-10 (IL-10) and transforming growth factor-β1 (TGF-β1) of goat PBMC. This membrane protein, together with TMEM63A, was also related to the regulation of galectin on phagocytosis and nitric oxide production of goat PBMC. However, it might not be involved in the regulation of galectin on the migration and interferon-γ (IFN-γ) transcription of goat PBMC.

**Conclusions:**

Our results showed that TMEM147 was a binding partner of Hco-gal-m/f and mediated the immunological regulation of Hco-gal-m/f on goat PBMC in a manner different to that of TMEM63A.

**Electronic supplementary material:**

The online version of this article (doi:10.1186/s13071-016-1640-0) contains supplementary material, which is available to authorized users.

## Background

*Haemonchus contortus* is a gastrointestinal parasitic nematode in ruminants (notably goats and sheep) that feeds on blood in the abomasum [1]. Infections with *H. contortus* can lead to anaemia, weight loss and death, and causes substantial economic losses to livestock production worldwide [2]. Galectins are an evolutionarily ancient family of proteins, closely related to carbohydrate-binding proteins, and located either intracellularly or extracellularly [3–6]. To date, 15 mammalian galectins (galectin-1 to 15) have been cloned and functionally characterized [7]. They can function as important immunological mediators of homeostasis and disease regulation, and display a remarkable functional diversity by participating in the regulation of cell differentiation, proliferation, migration, activation, apoptosis and cytokine production [4–6, 8].

Galectins can be produced by both the parasite and the host. Subsequent studies suggested that parasite galectin might play an important role in host-parasite interactions [9]. It was demonstrated that galectin-1 might mediate *Trichomonas vaginalis* adherence to human cervical epithelial cells by binding to *T. vaginalis* lipophosphoglycan (LPG) [10], and modulate macrophage apoptosis during infection with *Trypanosoma cruzi* [11]. Galectin-3 was reported to be involved in biological processes that affected the replication of *Plasmodium yoelii* [12, 13]*.* Furthermore, galectin-3 could significantly alter the pathogenic course of *Toxoplasma gondii* [14], *Trypanosoma cruzi* [15] and *Leishmania major* [16]. In additional to galectin-1 and galectin-3, other galectins are also involved in host-parasite interactions. For example, galectin-9 could recognize *L. major* by binding to the *L. major*-specific polygalactosyl epitope [17], and negatively regulate helper T cell 2-mediated (Th2-mediated) eosinophilic lung inflammation during infection with *Ascaris suum* [18]. Alternatively, transcription and expression of galectin-11 was increased in the abomasal mucosa following *Ostertagia ostertagi* infection [19]. Galectin-14 was identified in the mucosal wash from ovine abomasum following *Teladorsagia circumcincta* larval challenge [20], and was significantly negatively correlated with worm burden following a challenge infection with *H. contortus* third-stage larvae (L3s) [21].

In addition to the above functions, galectins from mammals could also bind to appropriate receptors on the cell surface and participate in a number of biological processes. It has been reported that binding of galectin-3 to mucin 1 (MUC1) could promote tumor cell malignancy [22] and the cell surface interaction of annexin A2 and galectin-3 could also modulate epidermal growth factor receptor signaling in epidermal growth factor receptor-2 (Her-2) negative breast cancer cells [23]. However, there are few reports regarding the binding partners of these galectins in the host. Therefore, research into the molecular mechanisms that govern the interactions between these galectins and host molecules will shed a new light on galectin-mediated immunomodulation.

In previous research, galectins of male *H. contortus* (Hco-gal-m) (Acc. No. AY253330) and galectins of female *H. contortus* (Hco-gal-f) (Acc. No. AY253331) were demonstrated to be two isoforms of galectin derived from male and female *H. contortus,* respectively [24]. It has also been suggested that recombinant Hco-gal-m/f (rHco-gal-m/f) could bind to the surface of goat PBMC *in vitro*, and induce biological effects, including promoting cell apoptosis and altering the transcription of interleukin-1β (IL-1β), interleukin-4 (IL-4), IL-10, TGF-β1, IFN-γ and tumor necrosis factor-α (TNF-α) mRNA [25–27]. Furthermore, transcriptional and proteomic analyses revealed that rHco-gal-m/f could modulate several signaling cascades in vitro, including the activation of vascular endothelial growth factor pathway, free radical producing pathway, nuclear factor-kappa B (NFkB) pathway and the ubiquitin-proteasome pathway [25].

In our previous study, TMEM63A of goat PBMC was identified to be a receptor of Hco-gal-m/f and knockdown of the *tmem63a* gene by RNAi altered cell proliferation, phagocytosis, nitric oxide production and cytokine mRNA transcription of goat PBMC induced by Hco-gal-m/f [27]. However, in the identification of binding partners for Hco-gal-m and Hco-gal-f by yeast two-hybrid (YTH) screening, it was found that TMEM147 and TMEM63A simultaneously bound to Hco-gal-m/f. However, the functions of TMEM147 in the regulation of Hco-gal-m/f on the PBMC remain unclear and whether TMEM147 is another receptor or partner protein of Hco-gal-m/f on goat PBMC is also unknown. In this study, the functions of TMEM147 in the regulation of *H. contortus* galectin on the goat PBMC were observed and the results showed that TMEM147 was another partner protein of Hco-gal-m/f. It mediated the functions of goat PBMC induced by Hco-gal-m/f differently with that of TMEM63A.

## Methods

### Animals and cells

Local crossbred goats (3–6 month-old) from the teaching and research flock at Nanjing Agricultural University were housed indoors in pens. They were fed with hay and whole shelled corn and provided with water *ad libitum*. All goats were dewormed twice at 2 weekly intervals with levamisole (8 mg/kg bodyweight) orally at the time of housing to remove naturally acquired strongylid infections [28]. According to standard parasitological techniques, after two weeks, the helminth eggs in fecal samples from each goat were detected by microscopy. Goats evincing no eggs were used in the subsequent study and the health was observed daily throughout the experiment. The isolation and culture of goat PBMCs and monocytes were performed as previously described [27].

### Identification of binding partners for Hco-gal-m and Hco-gal-f by yeast two-hybrid (YTH) screening

The identification of binding partners for Hco-gal-m/f was performed as previously described using YTH screening [27].

### Validation of the interaction between rHco-gal-m and TMEM147 by co-immunoprecipitation (co-IP) and immunoblotting

To validate the interaction between rHco-gal-m and candidate binding proteins, the forward IP and reverse IP experiments were performed independently as previously described [27]. The goat PBMC stimulated with rHco-gal-m for 12 h were pelleted and lysed. After preclearing treatment, triplicate 1 mg lysates were each incubated separately overnight at 4 °C with the following: rat anti-TMEM147-O immunoglobulin G (IgG) for input samples, rat anti-Hco-gal IgG for IP samples and normal rat IgG (Santa Cruz Biotechnology, Dallas, Texas, USA) for negative control samples in forward IP; rat anti-Hco-gal IgG for input samples, rat anti-TMEM147-O IgG for IP samples, normal rat IgG for negative control samples in reverse IP. Immune complexes were precipitated by 20 μl protein A/G PLUS-Agarose beads (Santa Cruz Biotechnology, Texas, USA) following the manufacturer’s protocol.

Immunoblotting assays were performed following co-IP according to the methods previously described [27]. The membrane was incubated with the respective primary antibodies: rat anti-TMEM147-O IgG for forward IP experiment or rat anti-Hco-gal IgG for reverse IP experiment overnight at 4 °C. Antibodies and the production of antibodies used in these experiments are described in Additional file 1: Protocols (Production of antibody); Additional file 2: Table S1; and Additional file 3: Figures S1–S4.

### Detection of the localization of TMEM147 in PBMC by immunofluorescence (IF)

The IF assay was performed according to previous research [27]. The fixed and permeabilized PBMC (10^5^ cells/sample) were incubated with 0.5 μg of the respective primary antibodies, rat anti-TMEM147-O IgG or negative rat IgG (for negative control) and incubated with the secondary antibody coupled to Cyanine dyes 3 (Cy3) fluorescent dye (Beyotime Biotechnology, Haimen, Jiangsu, China) (1:300). Cells were then stained with 5 μM 3,3'-Dioctadecyloxacarbocyanine (DiOC18(3); Beyotime Biotechnology, Haimen, Jiangsu, China) and 1.5 μM 2-(4-Amidinophenyl)-6-indolecarbamidine dihydrochloride (DAPI; Sigma, St. Louis, Missouri, USA), respectively, for 6 min each. Protein localization was observed with a 100× oil objective lens on a laser scanning confocal microscope (LSM710, Zeiss, Jena, Thuringia, Germany). Digital images were captured using the Zeiss microscope software package ZEN 2012 (Zeiss, Jena, Thuringia, Germany).

### Detection of the distribution of TMEM147 in T cells, B cells and monocytes in PBMC by flow cytometry

The distribution of TMEM147 in T cells and monocytes in PBMC were detected as previously described using flow cytometry [27]. PBMC (10^6^ cells/reaction), isolated as described above, were firstly stained with antibodies: 1 μg mouse anti-bovine cluster of differentiation 2-fluorescein isothiocyanate (CD2-FITC), 1 μg mouse anti-bovine cluster of differentiation 21-fluorescein isothiocyanate (CD21-FITC) and 1 μg mouse anti-bovine cluster of differentiation 14-fluorescein isothiocyanate (CD14-FITC) (AbDSerotec, Bio-Rad Laboratories, Hercules, California, USA), respectively, at 4 °C for 30 min. After permeabilization and washing, cells were then incubated with 1 μg rat anti-TMEM147-O IgG for 1 h and chicken anti-rat immunoglobulin G-phycoerythrin (IgG-PE) (1:300, Santa Cruz Biotechnology, Dallas, Texas, USA) for 30 min at room temperature. Normal mouse immunoglobulin G1-fluorescein isothiocyanate (IgG1-FITC) (Santa Cruz Biotechnology, Dallas, Texas, USA) and negative rat IgG (1 μg) were used to set a ‘fluorescence minus one’ control. Samples were examined on a FACS Calibur™ flow cytometer (BD Biosciences, San Jose, California, USA). Data were analyzed using FlowJo 7.6 software (Tree Star, Ashland, Oregon, USA).

### Small interfering RNA and cell treatment

Three small interfering RNAs (siRNA) were designed to knockdown the *tmem147* gene (Additional file 2: Table S2). TMEM147-siRNA-1, with the highest interference efficiency, was selected for further experiments (Additional file 1: Protocols (Transfection procedures for siRNA) and Additional file 3: Figure S5). The siRNAs used in this study were chemically synthesized by Invitrogen (Life Technologies, Shanghai, China) and dissolved in RNase-free water to 20 μM. The time required for knockdown was also determined (Additional file 1: Protocols (Transfection procedures for siRNA) and Additional file 3: Figure S5). The non-specific siRNA (ns siRNA) sequences used in the experiment are listed in Additional file 2: Table S2.

Cells were treated with siRNA transfection and then rHco-gal-m was added as stimulation. The concentration of rHco-gal-m (40 μg/ml) has been demonstrated to produce a distinct biological response without causing toxicity to the cells by previous dose response studies [26, 29, 30]. Cells for the RNAi period are shown as a blank group (group 1), ns siRNA group (group 2), ns siRNA/g group (group 3), 147-siRNA group (group 4) and 147-siRNA/g group (group 5), which were incubated with an equal volume of reduced serum medium (group 1), ns siRNA (groups 2 and 3) and TMEM147-siRNA-1 (groups 4 and 5), for 60 h at a concentration of 1 × 10^6^ cells/ml. rHco-gal-m in all RNAi groups (groups 3 and 5) was added 12 h before the end of the RNAi period [27].

### Cell proliferation assay

At the end of the RNAi period, cell proliferation assay of PBMC was performed as previously described using a cell counting kit-8 assay reagent (Beyotime Biotechnology, Haimen, Jiangsu, China) [27]. Cells in the blank group served as controls and the OD450 was set as 100 %. The cell proliferation index was calculated using the formula: OD450 group/OD450 control.

### Cell phagocytosis assay

Monocytes were collected at the end of the RNAi period and incubated with 1 mg/ml fluorescein isothiocyanate-dextran (FITC-dextran) (Sigma, St. Louis, Missouri, USA) in Roswell Park Memorial Institute 1640 (RPMI 1640; GIBCO, Grand Island, New York, USA) at 37 °C for 1 h [31]. The reaction was stopped using cold phosphate buffered saline (PBS) containing 2 % fetal bovine serum. Cells were washed three times and resuspended in PBS containing 2 % paraformaldehyde. The FITC-dextran internalization of monocytes was analyzed by flow cytometry (BD Biosciences, San Jose, California, USA). Data were analyzed using FlowJo 7.6 software (Tree Star, Ashland, Oregon, USA) and the cell phagocytosis index was calculated by considering the statistical data of Median Fluorescence Intensity (MFI) values in the blank group as 100 %.

### Measurement of nitric oxide (NO) production

According to a previous study using Total Nitric Oxide Assay Kit (Beyotime Biotechnology, Haimen, Jiangsu, China) [27], the NO production assay was performed by detecting intracellular nitrite in the PBMC with the Griess assay [32]. Absorbance was determined using a plate reader (Bio-Rad Laboratories, Hercules, California, USA) at 540 nm (OD540) and then converted to micromoles per liter using a standard curve generated by the addition of 0 to 80 μmol/l of sodium nitrite to fresh culture media.

### Cell migration assay

At the end of the RNAi period, the migration assay of PBMC was performed as previously described using Millicell® insert with 8.0 μm pores (Merck Millipore, Darmstadt, Hessen, Germany) [27]. The cells that migrated through the membrane into the lower chamber were determined with a Neubauer counting chamber. The results were presented as percentages of the seeded PBMC. Each experiment was performed in triplicate.

### Apoptosis assay

The Annexin V-FITC kit (Miltenyi Biotec, Bergisch Gladbach, Nordrhein-Westfalen, Germany) was used to estimate cell apoptosis, according to the manufacturer’s instructions. At the end of RNAi period, cells (1 × 10^6^ cells/ml) were harvested and used to check the apoptosis using the flow cytometer (BD Biosciences, San Jose, California, USA.). Data were analyzed using FlowJo 7.6 software (Tree Star, Ashland, Oregon, USA).

### Detection of cytokine transcription

40 μl of vehicle (PBS, unstimulated negative control) or rHco-gal-m at 1 μg/μl was added to the PBMC to yield a final volume of 1 ml per well and incubated for 12 h before the end of the RNAi period. The detection of cytokine transcription was performed as previously described by real-time PCR [27]. The reactions and conditions of real-time PCR are detailed in Additional file 1: Protocols (Real-time PCR reactions and conditions), and the primers for real-time PCR are listed in Additional file 2: Table S3. The amplification efficiencies of all targets and endogenous reference genes were verified to be similar by real-time PCR (Additional file 2: Table S3). Raw cycle thresholds (Ct), obtained from the ABI Prism 7500 software (version 2.0.6; Applied Biosystems, Foster City, California, USA), were used for the comparative Ct (2^-ΔΔCt^) method [33].

### Statistical analysis

Statistical analysis for significant differences was performed using the Graphpad Premier 6.0 software package (Graphpad Prism, San Diego, California, USA) at *P* < 0.001. Data were expressed as the mean ± the standard deviation (SD).

## Results

### TMEM147 is a novel binding protein for Hco-gal-m and -f

YTH screening assays were performed to find the binding partners of Hco-gal-m and -f from a goat PBMC cDNA library constructed previously [27]. Following two rounds of YTH screening, 20 clones encoding proteins were found to interact with the Hco-gal-m and -f proteins in yeast cells. Using DNA sequencing and BLASTn of GenBank, four genes products were identified. Besides TMEM63A, one of the products was TMEM147 (NCBI accession number JQ923484). Moreover, the amino acid sequences of TMEM147 were used to predict the transmembrane structure by TMHMM Server v.2.0 (http://www.cbs.dtu.dk/services/TMHMM/), and the results detailed in Additional file 3: Figure S4 indicate that TMEM147 has 7 transmembrane domains and largely resides within the membrane. As the same candidate proteins were identified using either Hco-gal-m or Hco-gal-f as bait, only rHco-gal-m was used in subsequent experiments as a representative protein [27, 28].

### Co-IP assays demonstrated that rHco-gal-m could bind to TMEM147

**Fig. 1:**
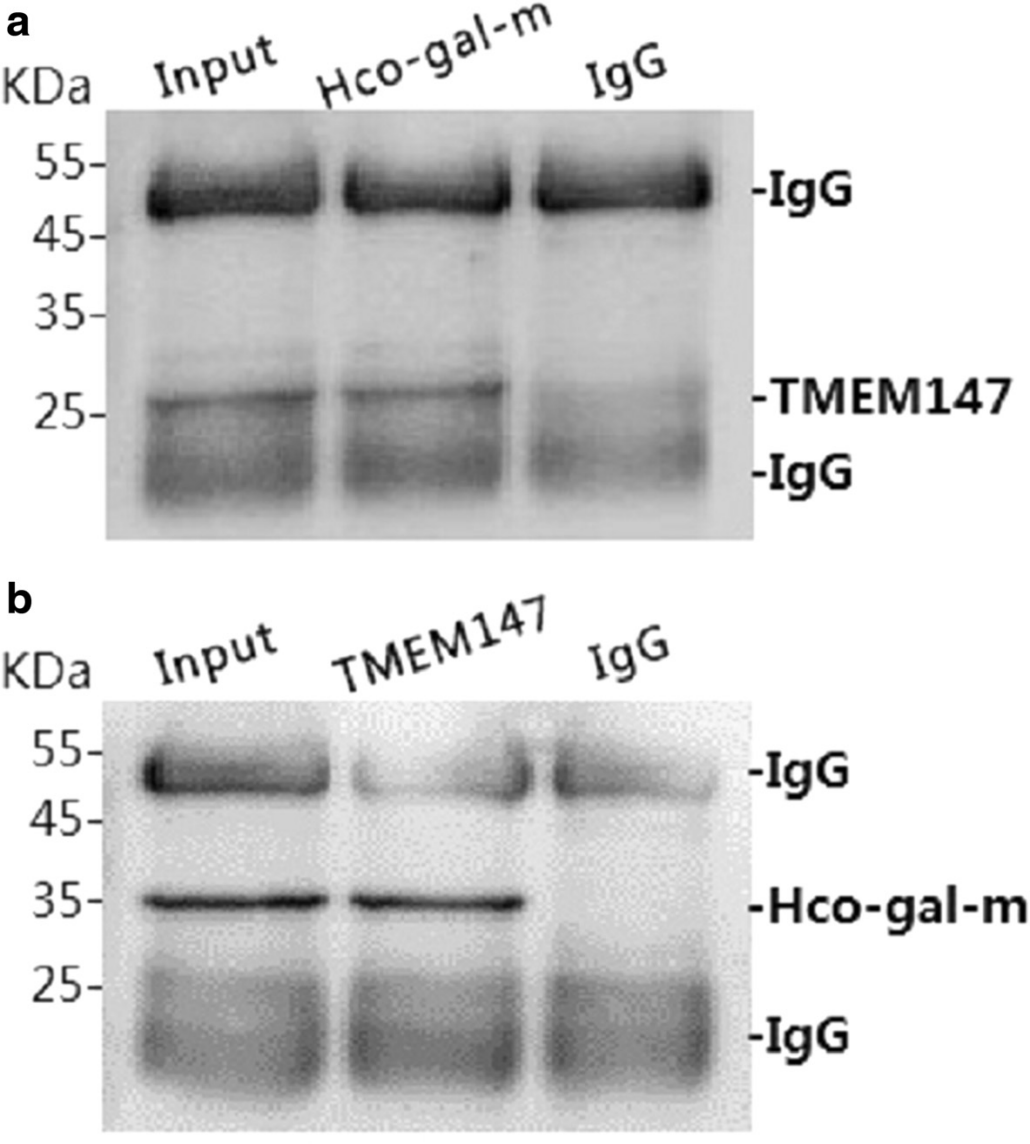
Co-IP assays indicate that rHco-gal-m can bind to TMEM147. Lane Input (**a**, **b**): Cell lysates were precipitated with rat anti-TMEM147-O IgG and rat anti-Hco-gal IgG. Lane IP (**a**, **b**): Cell lysates were precipitated with rat anti-Hco-gal IgG and rat anti-TMEM147-O IgG, respectively. Lane IgG (**a**, **b**): Cell lysates were precipitated with normal rat IgG. Immunoblot analysis using rat anti-TMEM147-O IgG and rat anti-Hco-gal IgG demonstrated that rHco-gal-m could bind to TMEM147. IP: immunoprecipitation

To confirm the results of YTH screening, two independent co-IP assays were performed in rHco-gal-m-stimulated (12 h) goat PBMC. After immunoblotting, TMEME147 were detected in rHco-gal-m immune complexes (IP) and in the PBMC lysates (Input), but not in the rat normal IgG control (IgG) group (Fig. 1a). Reciprocally, in the reverse co-IP assay, rHco-gal-m was detected in TMEM147 immune complexes (IP) and in the PBMC lysates (Input), but not in the rat normal IgG control (IgG) group (Fig. 1b). The results of the co-IP assays strongly indicated that the interactions of Hco-gal-m with TMEM147 in PBMC was the result of specific binding.

### TMEM147 was localized to the cell membrane and within the cell membrane in PBMC

**Fig. 2:**
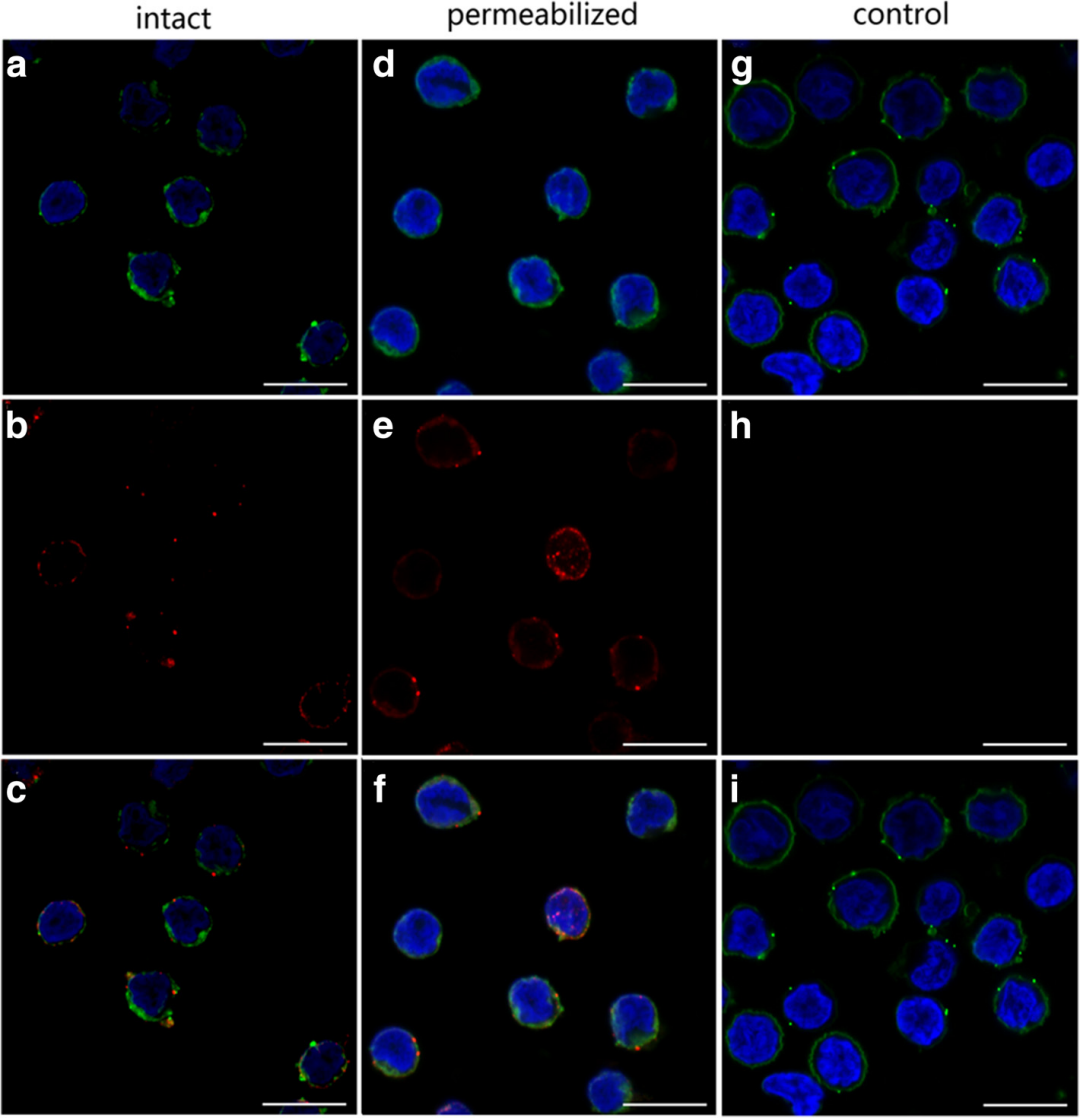
TMEM147 localized to the cell membrane and within the cell membrane in PBMC. Localization was carried out by incubation of cells with rat anti-TMEM147-O IgG (TMEM147) or negative rat IgG (Control). DIO (*green*), DAPI (*blue*) and Cy3-conjugated secondary antibodies (*red*) were utilized for triple staining. **a**, **d**, **g**: Cell membrane (*green*) and nuclei (*blue*) staining of cells. **b**, **e**, **h**: Staining of target proteins (*red*). **c**, **f**, **i**: A merged image of the three colors. TMEM147 localized to the cell membrane and within the cell membrane. *Scale-bars*: 10 μm

Using IF assay, the location of TMEM147 was detected in intact and permeabilized PBMC. Confocal microscopy images show that TMEM147 was found on the cell surface in intact cells (Fig. 2). However, TMEM147 could also be found in the cytosol of permeabilized cells (Fig. 2). In the control group, no red fluorescence was observed (Fig. 2).

### TMEM147 was expressed in T cells, B cells and monocytes of goat PBMC

**Fig. 3:**
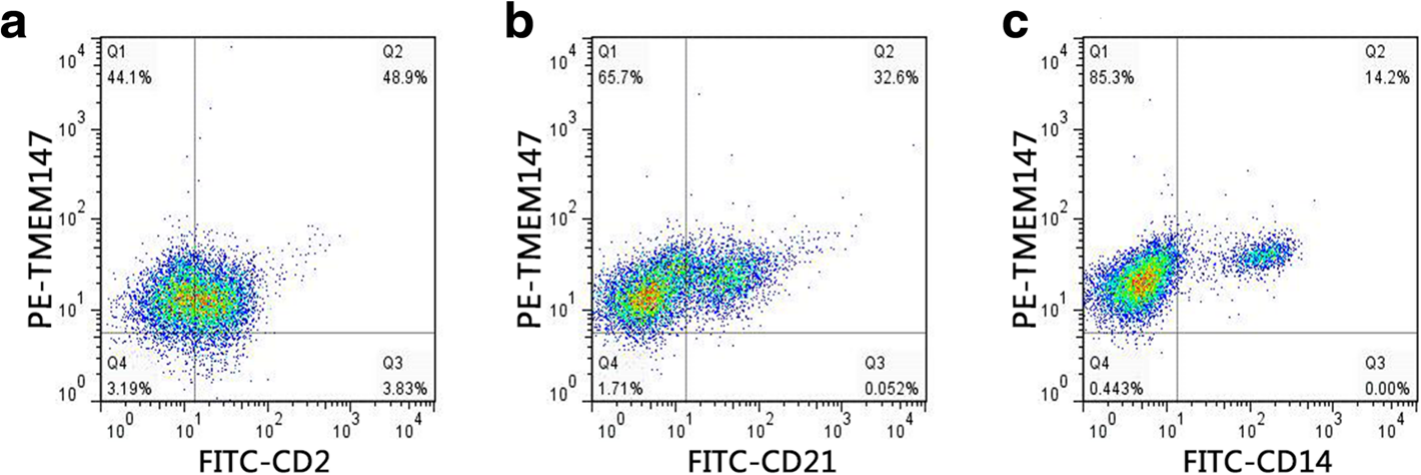
Analysis of TMEM147 expression in goat PBMC by flow cytometry. T cell (**a**), B cell (**b**) and monocyte (**c**) populations were identified using FITC-CD2, FITC-CD21 and FITC-CD14 (X-axis). **a** Q1: TMEM147^+^/CD2^−^; Q2: TMEM147^+^/CD2^+^; Q3: TMEM147^−^/CD2^+^; Q4: TMEM147^−^/CD2. **b** Q1:TMEM147^+^/CD21^−^; Q2: TMEM147^+^/CD21^+^; Q3: TMEM147^−^/CD21^+^; Q4: TMEM147^−^/CD21^−^. **c** Q1: TMEM147^+^/CD14^−^; Q2: TMEM147^+^/CD14^+^; Q3: TMEM147^−^/CD14^+^; Q4: TMEM147^−^/CD14^−^. The percentages of cells with different staining patterns are shown. The results presented here are representative of three independent experiments

In this study, the frequencies of TMEM147^+^ T cells (TMEM147^+^/CD2^+^) were 48.9 % and 3.8 % TMEM147^−^ T cells (TMEM147^−^/CD2^+^) (Fig. 3a). The frequencies of TMEM147^+^ B cells (TMEM147^+^/CD21^+^, 32.6 %) were approximately similar to the frequency of total B cells (CD21^+^, 32.6 % + 0.052 %, Fig. 3b). TMEM147^+^ monocytes (TMEM147^+^/CD14^+^, 14.2 %) were approximately 100 % of total monocytes (CD14^+^, 14.2 % + 0.00 %) in PBMC (Fig. 3c). These results indicate that the majority of goat PBMC expressed TMEM147.

### rHco-gal-m affected PBMC proliferation by the interaction with TMEM147

**Fig. 4:**
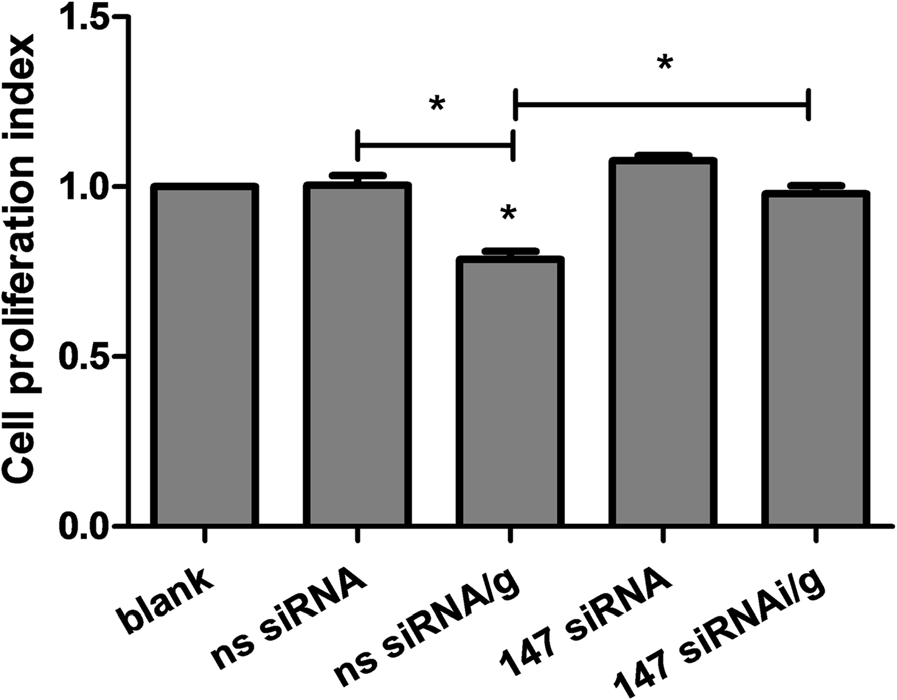
rHco-gal-m affected the PBMC proliferation by the interaction with TMEM147. Cell proliferation index was calculated considering the OD450 values in blank group as 100 %. Results presented here are representative of three independent experiments. Data are presented as the mean ± SD (*n* = 6); *P < 0.001 *versus* the ns siRNA group; an asterisk and a capped line designate two groups that differ significantly (*P* < 0.001)

Results showed that there was no PBMC multiplication in the ns siRNA group induced by the ns siRNA-treatment compared to the blank group (Fig. 4). Furthermore, PBMC proliferation in the ns siRNA/g group was significantly decreased (ANOVA, *F*_(4, 25)_ = 28.23, *P* < 0.0001) by rHco-gal-m compared to the ns siRNA group (Fig. 4). After TMEM147 siRNA-treatment, PBMC proliferation in the 147 siRNA/g group was significantly increased (ANOVA, *F*_(4, 25)_ = 28.23, *P* < 0.0001) compared to the ns siRNA/g group (Fig. 4). No significant difference (ANOVA, *F*_(4, 25)_ = 28.23, *P* = 0.0196) was induced by rHco-gal-m between the 147 siRNA/g and 147 siRNA groups. In addition, no significant difference (ANOVA, *F*_(4, 25)_ = 28.23, *P* = 0.1271) was observed between the ns siRNA and 147 siRNA groups (Fig. 4).

### rHco-gal-m affected monocytes phagocytosis by the interaction with TMEM147

**Fig. 5:**
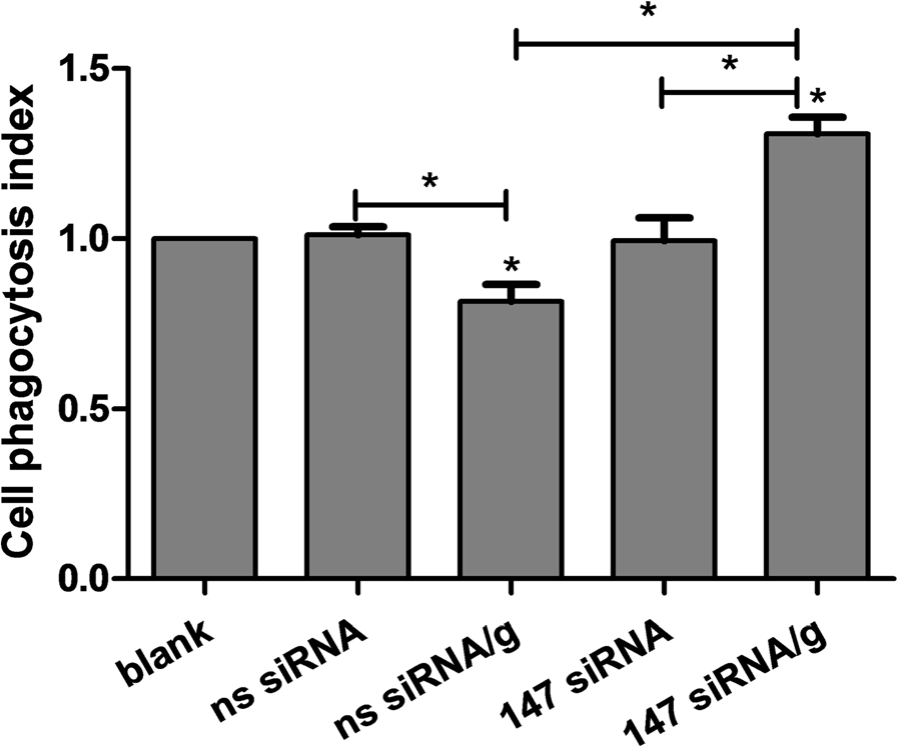
rHco-gal-m affected the monocytes phagocytosis by the interaction with TMEM147. Cell phagocytosis index was calculated considering statistic data of MFI (Median Fluorescence Intensity) values in blank group as 100 %. Results presented here are representative of three independent experiments. Data are presented as the mean ± SD (*n* = 4); *P < 0.001 *versus* the ns siRNA group; an asterisk and a capped line designate two groups that differ significantly (*P* < 0.001)

The cell phagocytosis assay was performed to explore the possible effects of TMEM147 and rHco-gal-m on monocyte phagocytosis in goat PBMC. After rHco-gal-m-treatment, monocyte phagocytosis in the ns siRNA/g group was significantly decreased (ANOVA, *F*_(4, 15)_ = 63.69, *P* = 0.0001) compared to the ns siRNA group (Fig. 5). However, monocyte phagocytosis in 147 siRNA/g was significantly increased compared to the 147 siRNA group (ANOVA, *F*_(4, 15)_ = 63.69, *P* < 0.0001) and ns siRNA/g group (ANOVA, *F*_(4, 15)_ = 63.69, *P* < 0.0001) (Fig. 5). No significant difference (ANOVA, *F*_(4, 15)_ = 63.69, *P* = 0.9789) was induced by TMEM147 siRNA-treatment between the 147 siRNA and ns siRNA groups (Fig. 5).

### rHco-gal-m affected PBMC nitric oxide production by the interaction with TMEM147

**Fig. 6:**
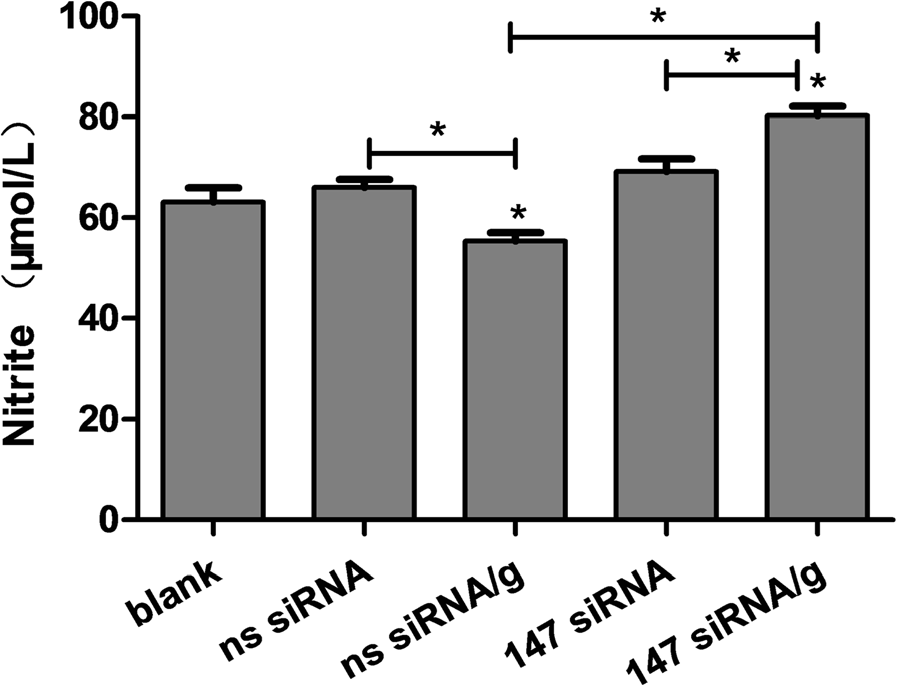
rHco-gal-m affected the PBMC nitric oxide production by the interaction with TMEM147. Results presented here are representative of three independent experiments. Data are presented as the mean ± SD (*n* = 4); *P < 0.001 *versus* the ns siRNA group; an asterisk and a capped line designate two groups that differ significantly (*P* < 0.001)

When PBMC were incubated with rHco-gal-m, nitric oxide production in the ns siRNA/g group was significantly suppressed (ANOVA, *F*_(4, 10)_ = 55.70, *P* = 0.0008) by rHco-gal-m compared to ns siRNA group (Fig. 6). After TMEM147 siRNA-treatments, there was no influence (ANOVA, *F*_(4, 10)_ = 55.70, *P* = 0.3917) on PBMC nitric oxide production in the 147 siRNA group compared with the ns siRNA group (Fig. 6), whilst the nitric oxide production in 147 siRNA/g was significantly increased (ANOVA, *F*_(4, 10)_ = 55.70, *P* = 0.0006) compared to the 147 siRNA group and ns siRNA/g group (Fig. 6).

### The interaction of rHco-gal-m with TMEM147 might not mediate PBMC migration

**Fig. 7:**
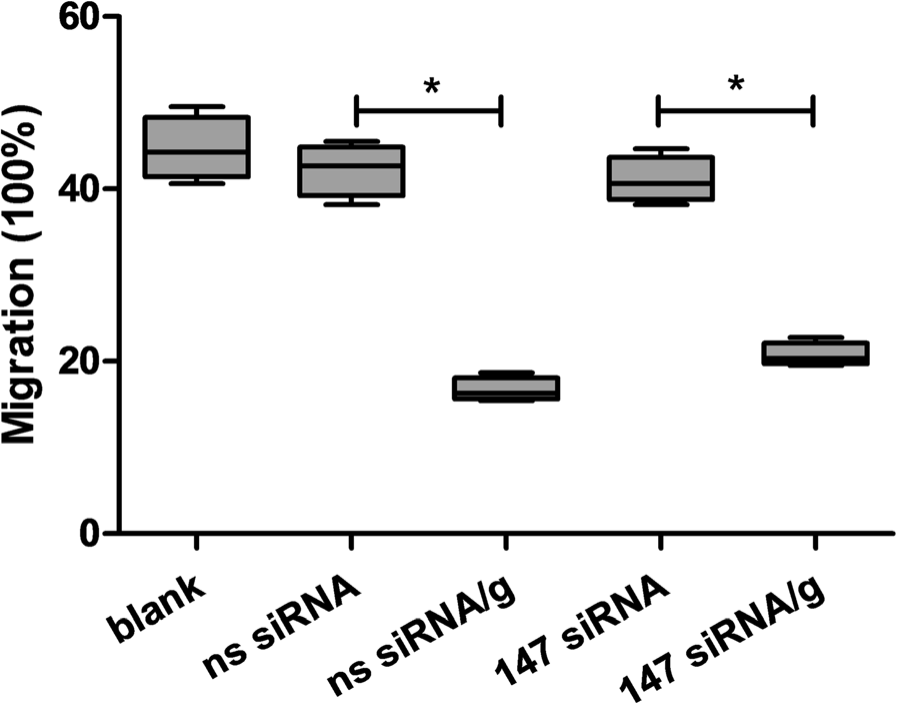
The interaction of rHco-gal-m with TMEM147 might not mediate the PBMC migration. The data are presented as box-and-whiskers plot, with the box containing 50 % of the values, and the whiskers showing the highest and the lowest values. The median is indicated by the horizontal bar, the mean value as square. Results presented here were collected from one independent experiment (*n* = 4) and are representative of three independent experiments; an asterisk and a capped line designates two groups that differ significantly (*P* < 0.001)

In order to explore the impact of TMEM147 knockdown and rHco-gal-m/f-treatment on PBMC migration, a cell migration assay was performed using Millicell® insert. No significant difference (ANOVA, *F*_(4, 15)_ = 103.3, *P* = 0.6838) was observed between the blank group (41.03 ± 2.69 %) and ns siRNA group (42.25 ± 3.04 %). Consistent with previous studies, the percentage of migrating PBMC in the ns siRNA/g group (16.66 ± 1.41 %) was significantly decreased (ANOVA, *F*_(4, 15)_ = 103.3, *P* < 0.0001) compared to the ns siRNA group. However, after TMEM147 siRNA-treatment, the decrease in 147 siRNA/g group (20.72 ± 1.41 %) was still prominent (ANOVA, *F*_(4, 15)_ = 103.3, *P* < 0.0001) compared to the 147 siRNA group (41.03 ± 2.69 %). No significant difference (ANOVA, *F*_(4, 15)_ = 103.3, *P* = 0.9620) between 147 siRNA and ns siRNA groups was induced by TMEM147 siRNA-treatment (Fig. 7). There was also no significant difference (ANOVA, *F*_(4, 15)_ = 103.3, *P* = 0.2322) between the 147 siRNA/g and ns siRNA/g groups induced by TMEM147 siRNA-treatment (Fig. 7).

### rHco-gal-m affected the apoptosis of goat PBMC by the interaction with TMEM147

**Fig. 8:**
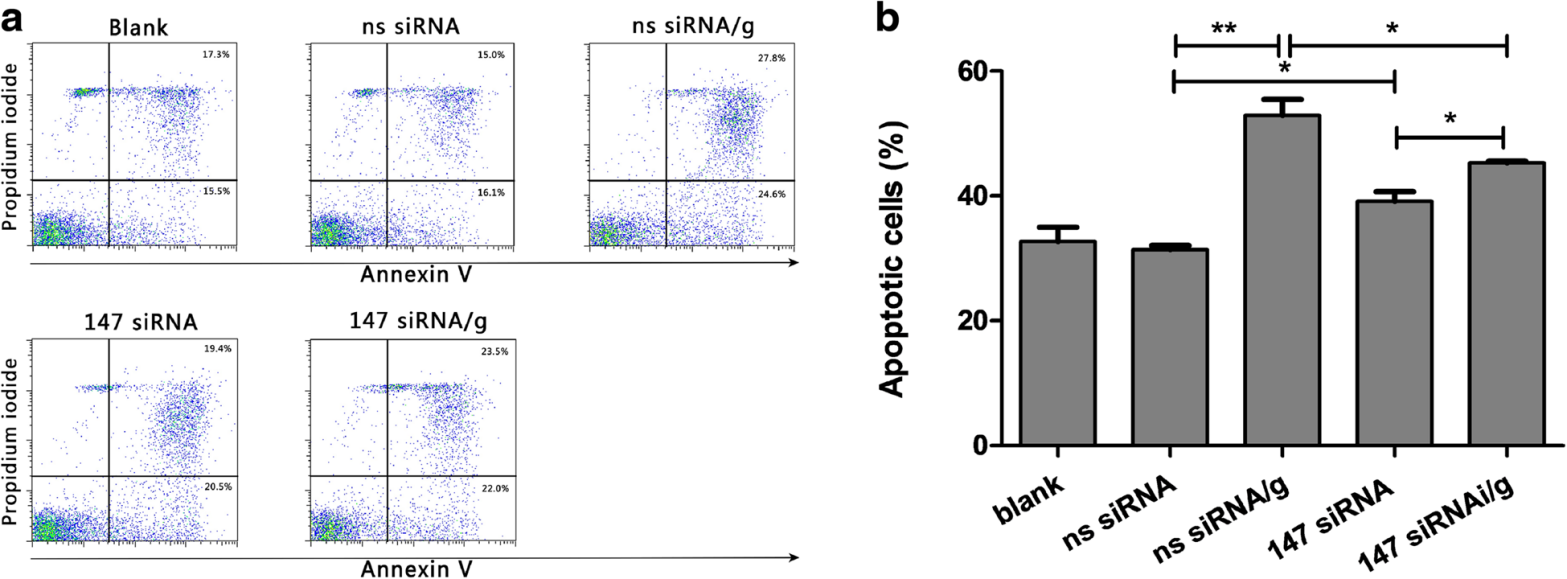
**a** Analysis of PBMC apoptosis by flow cytometry. Apoptosis of PBMC was determined by staining with annexin V and PI followed by flow cytometric analysis. The percentages of cells with different staining patterns are shown. The results presented here are representative of three independent experiments. **b** rHco-gal-m affected the apoptosis of goat PBMC by the interaction with TMEM147. The percentage of apoptosis was measured on four separate occasions. Results presented here are representative of three independent experiments. Data are presented as the mean ± SD (*n* = 3); an asterisk and a capped line designate two groups that differ significantly (*P* < 0.01); double asterisk and a capped line designates two groups that differ highly significantly (*P* < 0.001)

To explore the impact of TMEM147 knockdown and rHco-gal-m/f-treatment on PBMC apoptosis, a cell apoptosis assay was performed. The externalization of membrane phosphatidylserine (PS) was used as a marker of cell apoptosis and the positive DNA staining was used as an indicator of membrane leakage. No significant change (ANOVA, *F*_(4, 10)_ = 80.93, *P* = 0.8863) on apoptosis was observed between the blank and ns siRNA groups (Fig. 8). The percentage of apoptotic cells in the ns siRNA/g group was dramatically augmented (ANOVA, *F*_(4, 10)_ = 80.93, *P* < 0.0001) by rHco-gal-m compared to the ns siRNA group (Fig. 8). After knockdown of the *tmem147* gene, the apoptosis induced by rHco-gal-m in the 147 siRNA/g group was significantly suppressed (ANOVA, *F*_(4, 10)_ = 80.93, *P* = 0.0023) by TMEM147 siRNA-treatment compared to the ns siRNA/g group (Fig. 8). Moreover, the increase of apoptotic cells induced by rHco-gal-m between the 147 siRNA and 147 siRNA/g groups was diminished in TMEM147 siRNA-treated cells (Fig. 8). Meanwhile, there was a significant increase (ANOVA, *F*_(4, 10)_ = 80.93, *P* = 0.0020) between the ns siRNA and the 147 siRNA groups (Fig. 8).

### The interaction of rHco-gal-m with TMEM147 affected the transcription of cytokines in goat PBMC

**Fig. 9:**
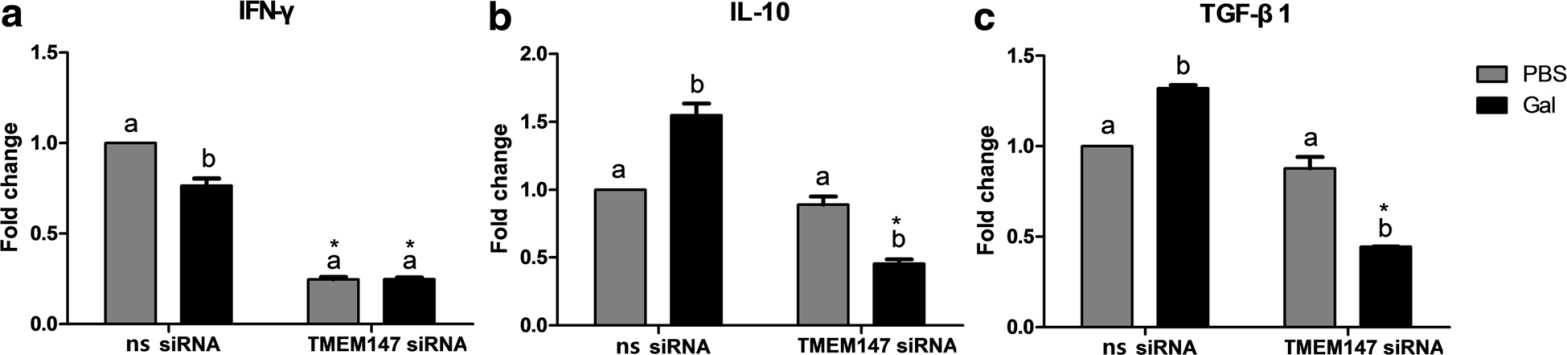
Relative levels of cytokine mRNA transcripts in goat PBMC pretreated with negative or TMEM147 siRNA. Goat PBMC pretreated with TMEM147 siRNA were stimulated by PBS (vehicle) or rHco-gal-m. **a** IFN-γ. **b** IL-10. **c** TGF-β1. Data are presented as the mean ± SD (*n* = 3). Significant differences between the two stimulation conditions for each RNAi group are indicated by different lower case letters (*P* < 0.001). The asterisk (*) designates that the mean of the TMEM147 knockdown group differs significantly from the mean of the negative siRNA group (*P* < 0.001) for a stimulation condition. Results presented here are representative of three independent experiments

After treatments by ns or TMEM147 siRNA, the goat PBMC were stimulated by PBS (vehicle) or rHco-gal-m. Real-time PCR analyses demonstrated that the transcription of IL-10 (ANOVA, *F*_(3, 8)_ = 199.2, *P* < 0.0001) and TGF-β1 (ANOVA, *F*_(3, 8)_ = 364.3, *P* < 0.0001) in non-specific siRNA-treated cells were significantly increased by rHco-gal-m (Fig. 9b, c), whilst the transcription of IFN-γ in non-specific siRNA-treated cells was prominently suppressed (ANOVA, *F*_(3, 8)_ = 949.5, *P* < 0.0001) by rHco-gal-m (Fig. 9a). Exposure of TMEM147 siRNA-treated cells to rHco-gal-m significantly decreased transcription of IL-10 (ANOVA, *F*_(3, 8)_ = 199.2, *P* < 0.0001) and TGF-β1 (ANOVA, *F*_(3, 8)_ = 364.3, *P* < 0.0001) mRNA, whilst transcription of IFN-γ in the cells was not affected (ANOVA, *F*_(3, 8)_ = 949.5, *P* = 0.9994).

Transcription of IL-10 (ANOVA, *F*_(3, 8)_ = 199.2, *P* < 0.0001) and TGF-β1 (ANOVA, *F*_(3, 8)_ = 364.3, *P* < 0.0001) in rHco-gal-m-treated cells were significantly diminished by TMEM147 siRNA-treatment, but unaffected (ANOVA, *F*_(3, 8)_ = 199.2, *P* = 0.1505 for IL-10; *F*_(3, 8)_ = 364.3, *P* = 0.0080 for TGF-β1) in PBS-treated cells (Fig. 9b, c). Notably, the transcription of IFN-γ in TMEM147 siRNA group were all prominently decreased (ANOVA, *F*_(3, 8)_ = 949.5, *P* < 0.0001) compared to the non-specific siRNA group by the same stimuli (Fig. 9a).

## Discussion

TMEM147 is a highly conserved membrane protein amongst mammals and widely expressed in many peripheral and central tissues whose function is still being elucidated. Rosemond et al. [34] reported that TMEM147 is a binding protein of M3R M3 muscarinic acetylcholine receptor and might act as a potent negative regulator of M3R function by changing the stimulatory effects of carbachol on H508 human colon cancer cell proliferation and p90 ribosomal S Kinase (p90RSK) activation. Dettmer et al. [35] suggested that TMEM147 was also a binding partner of a membrane protein complex, the Nicastrin-like protein-Nodal modulator (Nicalin-NOMO) complex. Nodal factors can initiate signaling cascades and drive the transcription of specific genes which affect cell proliferation [36]. Combined, these indicate that TMEM147 may have important functional roles in physiological processes. In this study, YTH screening and co-IP assays demonstrated that TMEM147 could bind to Hco-gal-m and Hco-gal-f. After knockdown of the *tmem147* gene by RNAi, the influence of rHco-gal-m on proliferation, phagocytosis, nitric oxide production, apoptosis and cytokine expression of the siRNA-treated cells were all changed. This indicated that TMEM147 mediated the regulation of rHco-gal-m on the goat PBMC.

Rosemond et al. [34] and Dettmer et al. [35] reported that TMEM147 localized to the endoplasmic reticulum (ER) membranes of co-transfected mammalian cells (COS-7 cells) and was enriched in ER membranes in HeLa cells. However, in this study, it was found that TMEM147 localized to both the cell surface and the inside of cells (Fig. 2). It was therefore suspected that TMEM147 might have different localizations in different kinds of cells. Our data suggest that TMEM147 was expressed by all B cells, monocytes and most T cells in goat PBMC (Fig. 3). Wang et al. [28] reported that different PBMC subsets had distinct responses to stimulation of rHco-gal-m/f. Hence, the specific functions of TMEM147 expressed by specific cell subsets and the nature and functions of these TMEM147-negative cells needs to be further investigated.

It has been suggested that TMEM147 was implicated in the regulation of cell proliferation [34, 35], and rHco-gal-m inhibited the proliferation of T cells in goat PBMC in vitro [27, 28]. In this study, it was found that the co-incubation with rHco-gal-m significantly suppressed the PBMC proliferation in the ns siRNA-treated cells, while the PBMC proliferation in rHco-gal-m-treated cells was significantly increased after TMEM147 siRNA-treatment (Fig. 4). It indicated that TMEM147 played a key role in the regulation of rHco-gal-m on the PBMC proliferation. In a previous study, TMEM63A specific siRNA-treatment could not effectively reverse the inhibition of rHco-gal-m on the PBMC proliferation [27]. So the modulation of cell proliferation in goat PBMC by rHco-gal-m was mainly mediated by TMEM147, but not TMEM63A.

It was demonstrated that phagocytosis could make a contribution to the clearance of damaged cells and host defense against pathogens, and a number of galectin family members have been shown to play an important role in phagocytosis [37]. In this study, the phagocytosis of monocytes in the ns siRNA/g group was significantly suppressed by rHco-gal-m (Fig. 5). After TMEM147 siRNA-treatment, the phagocytosis of monocytes in the 147 siRNA/g group was significantly increased compared to the 147 siRNA group and ns siRNA/g group (Fig. 5). It is therefore expected that the interaction between Hco-gal-m and TMEM147 plays an important role in the modulation of cell phagocytosis. In previous study [27], it was shown that the effective potency of TMEM63A was the same to TMEM147. However, the real functions of these two proteins or their collaboration in the regulation of phagocytosis still needs further research.

Nitric oxide (NO) has been implicated in host non-specific defence against a variety of infections and play roles in a number of parasitic infections such as malaria, toxoplasmosis, leishmaniosis, trypanosomosis and schistosomosis [38]. In this study, nitric oxide production in 147 siRNA/g group was significantly increased compared to the 147 siRNA group and ns siRNA/g group (Fig. 6). In the study of TMEM63A, the nitric oxide production in the 63A siRNA/g group was significantly increased compared to the ns siRNA/g group, while the production in 63A siRNA/g group was still significantly lower than that in the 63A siRNA group [27]. These findings revealed that rHco-gal-m inhibited the nitric oxide production by interactions with TMEM147 and TMEM63A. However, TMEM147 might play a greater role than TMEM63A in the rHco-gal-m-mediated regulation of nitric oxide production on goat PBMC.

It was demonstrated that rHco-gal-m/f significantly decreased the ability of cell migration in goat PBMC [25]. This description was further supported by our data. However, the significant decrease induced by rHco-gal-m was not changed by the TMEM147 siRNA-treatment (Fig. 7). In the study of TMEM63A, the suppression of cell migration induced by rHco-gal-m in the ns siRNA/g group was significantly changed by TMEM63A siRNA-treatment [27]. These results indicated that it was TMEM63A, not TMEM147, involved in the rHco-gal-m-mediated regulation of cell migration on goat PBMC.

It was reported that many members of galectin family were implicated in the regulation of cell apoptosis [39–41], and rHco-gal-m could induce cell apoptosis in goat PBMC [26, 28]. In this study, TMEM147 siRNA-treatment could significantly decrease cell apoptosis induced by rHco-gal-m (Fig. 8). Our data strongly suggest that TMEM147 was involved in the regulation of PBMC apoptosis induced by rHco-gal-m.

Cytokines play an important role in regulating development, differentiation and expression of functional effecters of the immune system, and the profile of immune reactions depends on cytokine contents at the recognition of parasite [42–45]. Recent studies reported that galectin-1 could affect the Th1/Th2 balance by mediating Th1 cell apoptosis and stimulating Th2 cytokine secretion [46], and IL-10 played important roles in mediating the immunosuppressive activities of galectin-1 [47]. Our previous studies demonstrated that rHco-gal-m increased the mRNA transcript levels of IL-10 and TGF-β1, whilst decreasing the transcription levels of IFN-γ [27, 29]. In this study, TMEM147 siRNA-treatment significantly decreased the transcript levels of IFN-γ either in the rHco-gal-m treated group or in the PBS control group (Fig. 9). In the study of TMEM63A, TMEM63A siRNA-treatment could significantly enhance the transcription of IFN-γ in both rHco-gal-m treated and PBS control groups [27]. It was suggested that the inhibition of IFN-γ transcription induced by rHco-gal-m was mainly mediated by TMEM63A. The results also showed that the enhancement of IL-10 and TGF-β1 transcription induced by rHco-gal-m in the ns siRNA groups were reversed by TMEM147 siRNA-treatment (Fig. 9), while slightly decreased by TMEM63A siRNA-treatment [27]. These indicated that the rHco-gal-m enhanced the transcription of IL-10 and TGF-β1 mainly through the interaction with TMEM147. The real regulation mechanisms of cytokines requires further research.

## Conclusions

Our data strongly suggest that TMEM147 is a binding partner or a membrane receptor for Hco-gal-m/f in goat PBMC. The interaction of galectin with TMEM147 mainly mediated cell proliferation, cell apoptosis, transcription of IL-10 and TGF-β1 of goat PBMC. This membrane protein, together with TMEM63A, was also related to the regulation of galectin on phagocytosis and nitric oxide production of goat PBMC. It is worth noting that TMEM63A might play a greater role than TMEM147 in the regulation of galectin in the migration and IFN-γ transcription of goat PBMC. These findings delivered new clues to the elucidation of the mechanisms involved in immune evasion by nematodes and in parasite-host interactions. However, the detailed functions of TMEM147 and TMEM63A in the regulation of galectin on the goat PBMC are still needed for further research.

## Abbreviations

CD14-FITC, cluster of differentiation 14-fluorescein isothiocyanate; CD21-FITC, cluster of differentiation 21-fluorescein isothiocyanate; CD2-FITC, cluster of differentiation 2-fluorescein isothiocyanate; co-IP, co-immunoprecipitation; Cy3, Cyanine dyes 3; DAPI, 2-(4-Amidinophenyl)-6-indolecarbamidine dihydrochloride; DiOC18(3), 3,3'′-Dioctadecyloxacarbocyanine; Hco-gal-m/f, galectins of male and female *Haemonchus contortus*; Her-2, epidermal growth factor receptor-2; IFN-γ, interferon-γ; IgG, immunoglobulin G; IgG-PE, immunoglobulin G-phycoerythrin; IL-10, interleukin-10; IL-1β, interleukin-1β; IL-4, interleukin-4; L3s, third-stage larvae; LPG, Lipophosphoglycan; MFI, Median Fluorescence Intensity; MUC1, mucin 1; NFkB, nuclear factor-kappa B; Nicalin-NOMO, Nicastrin-like protein-Nodal modulator; Ns, non-specific; p90RSK, p90 ribosomal S Kinase; PBMC, peripheral blood mononuclear cells; PBS, phosphate buffered saline; rHco-gal-m, recombinant galectins of male *Haemonchus contortus*; rHco-gal-m/f, recombinant galectins of male and female *Haemonchus contortus*; RNAi, RNA interference; siRNA, small interfering RNA; TGF-β1, transforming growth factor-β1; Th1, helper T cell 1; Th2, helper T cell 2; TMEM147, transmembrane protein 147; TMEM63A, transmembrane protein 63A; TNF-α, tumor necrosis factor-α; YTH, yeast two-hybrid.

## Additional files

Additional file 1: Protocols. (DOCX 20 kb) Additional file 2: Table S1. Primer sequences for PCR amplification. **Table S2.** siRNA sequences for gene knockdown. **Table S3.** Primer sequences for real-time PCR. (DOCX 14 kb) Additional file 3: Figure S1. N-terminal signal peptide prediction. **Figure S2.** Purification of recombinant TMEM147 and Hco-gal-m. **Figure S3.** Confirmation of polyclonal antibody specificity by western blot. **Figure S4.** Membrane protein prediction using TMHMM Server v.2.0. **Figure S5.** The knockdown efficiency of TMEM147 at different time points. (DOCX 1555 kb)
