# Supplementary material for: Transmembrane protein 147 (TMEM147): another partner protein of Haemonchus contortus galectin on the goat peripheral blood mononuclear cells (PBMC)
Source: Parasit Vectors. 2016 Jun 23;9:355. doi: 10.1186/s13071-016-1640-0 (PMC4918192; doi:10.1186/s13071-016-1640-0)
Supplement: Additional file 1: — Protocols. (DOCX 20 kb) [file 13071_2016_1640_MOESM1_ESM.docx]

**Protocols**

**1 Expression of recombinant proteins**

No antibody was available, so polyclonal antibodies were required. The polyclonal antibodies were generated using recombinant protein as antigen. The fragment encoding Hco-gal-m was amplified by PCR from the recombinant plasmid pBV220-Hco-gal-m which was constructed previously in our laboratory [[1](#_ENREF_1)].

TMEM147 was predicted to represent integral membrane proteins; full-length forms of TMEM147 cannot be readily expressed in *E. coli* expression systems. A partial region of TMEM147 was chosen for expression based on our prediction of membrane proteins using TMHMM Server v.2.0 (http://www.cbs.dtu.dk/services/TMHMM/; Additional file 6: Figure S4). Using cDNA from goat PBMC, the partial insert fragment of TMEM147 was amplified by PCR as previous description. Primer sequences for PCR amplification are listed in the supporting information (Additional file 2: Table S1).

After confirming the sequence of the PCR product, TMEM147 and Hco-gal-m fragments were extracted for ligation into the pET-28a(+) vector (Merck, Darmstadt, Hessen, Germany). Following ligation, the constructed plasmids were transformed into *E. coli* strain BL21 (DE3). Recombinant proteins were induced in cells using 1 mM isopropyl-β-d-thiogalactoside (IPTG) treatment. Proteins with His-tags were then purified using a fast protein liquid chromatograph equipped with an AKTA FPLC system (GE Healthcare, Chalfont St. Giles, Buckinghamshire, UK). SDS-PAGE gels were used to confirm the size and purity of the recombinant proteins (Additional file 4: Figure S2). Thrombin Kits (Merck, Darmstadt, Hessen, Germany) were used to cleave recombinant fusion proteins. After removing recombinant fusion proteins, lipopolysaccharide (LPS) was depleted from the proteins using the Detoxi-Gel Affinity Pak prepacked columns (Pierce Biotechnology Inc., Rockford, Illinois, USA), in accordance with the manufacturer’s instructions. Protein concentrations were determined using the Pierce^TM^ BCA^TM^ Protein Assay Kit (Thermo Fisher Scientific, Waltham, Massachusetts, USA) and were then diluted to 1 mg/mL using PBS. Endotoxin concentrations were measured using the LAL gel clot assay (Associates of Cape Cod Inc., East Falmouth, Massachusetts, USA), which showed that endotoxin concentrations for all recombinant proteins were less than 0.25 EU/mL. Ample aliquots were stored at –70 °C until use.

**2 Production of antibody**

SD rats (body weight, ~150 g) were purchased from the Experimental Animal Center of Jiangsu, PR China (Qualified Certificate: SCXK 2008-0004) and were raised in a sterilized room and fed sterilized food and water.

The rats were immunized with 100 μg protein four times at 15-day intervals. Negative serum was collected before the first immunization. The polyclonal antisera were collected 7 days after the final immunization. Rat antisera were subjected to 50% ammonium sulfate precipitation, using a published technique [[2](#_ENREF_2)]. The precipitated fraction was re-suspended in PBS (pH 7.4) for 24 h at 4°C, including three changes of buffer during this time period. PD MidiTrap G-25 (GE Healthcare, Pittsburgh, PA, USA) was used to desalt and adjust this crude preparation to the optimal pH and ionic binding buffer strength (20 mM sodium phosphate, pH 7.0) for IgG purification. Rat IgG was then purified using HiTrap Protein G HP (GE Healthcare, Chalfont St. Giles, Buckinghamshire, UK) following the manufacturer’s instructions. Rat IgG concentrations were determined using the Pierce^TM^ BCA^TM^ Protein Assay Kit (Thermo Fisher Scientific, Waltham, Massachusetts, USA). Purified rat IgGs, including preimmune IgG and specific IgG, were each diluted to 0.5 mg/mL. Then, IgG aliquots were stored at –70 °C until use. The specificities of IgGs were determined by immunoblotting (Additional file 5: Figure S3). The binding specificity of the antibody was further investigated and confirmed by mass-spectometry.

**3 Transfection procedures for siRNA**

For each siRNA transfection, 1×10^6^ cells/well were cultured in 12-well plates. The cells were transfected with 40 pmol TMEM63A-siRNA-1 along with 2 μL Lipofectamine^®^ RNAiMAX Reagent (Life Technologies, Shanghai, USA). In the negative control group, cells were transfected with 40 pmol non-specific siRNA (ns siRNA) and 2 μL Lipofectamine® RNAiMAX Reagent [[3](#_ENREF_3)].

To determine the optimal time to achieve maximal interference, cells were harvested at 24, 48 and 60 h after siRNA transfection. Cells were split to two aliquots, one aliquot was lysed using lysis buffer for western blot analysis, and the other aliquot was used for total RNA extraction to confirm the knockdown of target genes using RT-PCR analysis. The mRNA expression levels of beta-actin were used as an internal control and the stability of beta-actin expression was validated. The mouse monoclonal antibodies against beta-actin (1:200) and chicken anti-mouse IgG-HRP (1:3000) were purchased from Santa Cruz Biotechnology (Santa Cruz Biotechnology, Dallas, Texas, USA). Each experiment was performed in triplicate.

**4 The real-time PCR reactions and conditions**

The real-time PCR reactions were carried out in 96-well optical reaction plates (Bio-Rad Laboratories , Hercules，California，USA) with 200 nM each specific primer, 2 μl cDNA (20 ng, as described in the experimental procedures), and SYBR® Green Supermix (Bio-Rad Laboratories , Hercules，California，USA) using an ABI 7500 Real-Time PCR system (Applied Biosystems, Foster City, California, USA). The PCR conditions were as follows: 95°C for 30 sec, followed by 95°C for 15 sec, 60°C for 30 sec and 72°C for 30 sec for 40 cycles.

**Supplemental References**

1. Beck BM, Rice CD: **Serum antibody levels against select bacterial pathogens in Atlantic bottlenose dolphins, Tursiops truncatus, from Beaufort NC USA and Charleston Harbor, Charleston, SC, USA**. *Marine environmental research* 2003, **55**(2):161-179.

2. Yanming S, Ruofeng Y, Muleke CI, Guangwei Z, Lixin X, Xiangrui L: **Vaccination of goats with recombinant galectin antigen induces partial protection against Haemonchus contortus infection**. *Parasite immunology* 2007, **29**(6):319-326.

3. Yuan C, Zhang H, Wang W, Li Y, Yan RF, Xu LX, Song XK, Li XR: **Transmembrane protein 63A is a partner protein of Haemonchus contortus galectin in the regulation of goat peripheral blood mononuclear cells**. *Parasite Vector* 2015, **8**.
