# Supplementary material for: Transmembrane protein 147 (TMEM147): another partner protein of Haemonchus contortus galectin on the goat peripheral blood mononuclear cells (PBMC)
Source: Parasit Vectors. 2016 Jun 23;9:355. doi: 10.1186/s13071-016-1640-0 (PMC4918192; doi:10.1186/s13071-016-1640-0)
Supplement: Additional file 2: Table S1. — Primer sequences for PCR amplification. Table S2. siRNA sequences for gene knockdown. Table S3. Primer sequences for real-time PCR. (DOCX 14 kb) [file 13071_2016_1640_MOESM2_ESM.docx]

**Supporting Tables**

**Table S1. Primer sequences for PCR amplification**

| Gene Name | Primer Sequence (5’-3’) |
| --- | --- |
| Hco-Gal-m | GCGgaattcATGGTGTCACAGTTCCTAC (*Eco*R I) |
|  | TATgtcgacCTACTGGATCTGGATGCC (*Sal* I) |
| TMEM-147 | TTGgaattcTTCTTTCCCACCTGGGAA (*Eco*R I) |
|  | CAGctcgagAACCATGATCTTGTATTCCCC (*Xho* I) |

**Table S2. siRNA sequences for gene knockdown**

| Gene Name | Primer Sequence (5’-3’) |
| --- | --- |
| TMEM147-siRNA-1 | GCAGACCUGAUAGGCCUAATT |
|  | UUAGGCCUAUCAGGUCUGCTT |
| TMEM147-siRNA-2 | CCUUGUCAUGUCCCGGAAUTT |
|  | AUUCCGGGACAUGACAAGGTT |
| TMEM147-siRNA-3 | CCUUUGUCAUGGAGACCUUTT |
|  | AAGGUCUCCAUGACAAAGGTT |
| Non-specific siRNA | UUCUCCGAACGUGUCACGUTT |
|  | ACGUGACACGUUCGGAGAATT |

**Table S3. Primer sequences for real-time PCR**

| Gene Name | | Primer Sequence (5’-3’) | Amplification efficiency (%)* | Correlation coefficients (r^2^) |
| --- | --- | --- | --- | --- |
| beta-actin | | CACCACACCTTCTACAAC | 95.41 | 0.9991 |
|  | TCTGGGTCATCTTCTCAC | |  |  |
| TMEM147 | | CGAGAAGCACAGCAAGAATATCAC | 95.5 | 0.9992 |
|  | | GCCAATAACCAGGGTCCTACAG |  |  |
| IL-10 | CCTTGTCGGAAATGATCCAG | | 98.68 | 0.9993 |
|  | AGGGCAGAAAACGATGACAG | |  |  |
| IFN-γ | GAACGGCAGCTCTGAGAAAC | | 98.02 | 0.9982 |
|  | GGTTAGATTTTGGCGACAGG | |  |  |
| TGF-β1 | CATGAACCGGCCCTTCCT | | 98.98 | 0.9996 |
|  | GAAGTCAATGTAGAGCTGACGAACA | |  |  |

^*^ Amplification efficiency (%) = (10^-1/slope^ -1) ×100
