# Supplementary material for: Transmembrane protein 147 (TMEM147): another partner protein of Haemonchus contortus galectin on the goat peripheral blood mononuclear cells (PBMC)
Source: Parasit Vectors. 2016 Jun 23;9:355. doi: 10.1186/s13071-016-1640-0 (PMC4918192; doi:10.1186/s13071-016-1640-0)

**Figures**

**Figure S1. N-terminal signal peptide prediction.** The amino acid sequences of TMEM147 and Hco-gal-m (NCBI accession numbers: JQ923484 and AY253330) were used to predict N-terminal signal peptides by SignalP 4.1 Server. a, TMEM147; b, Hco-gal-m. No protein encoded a predicted N-terminal signal peptide.


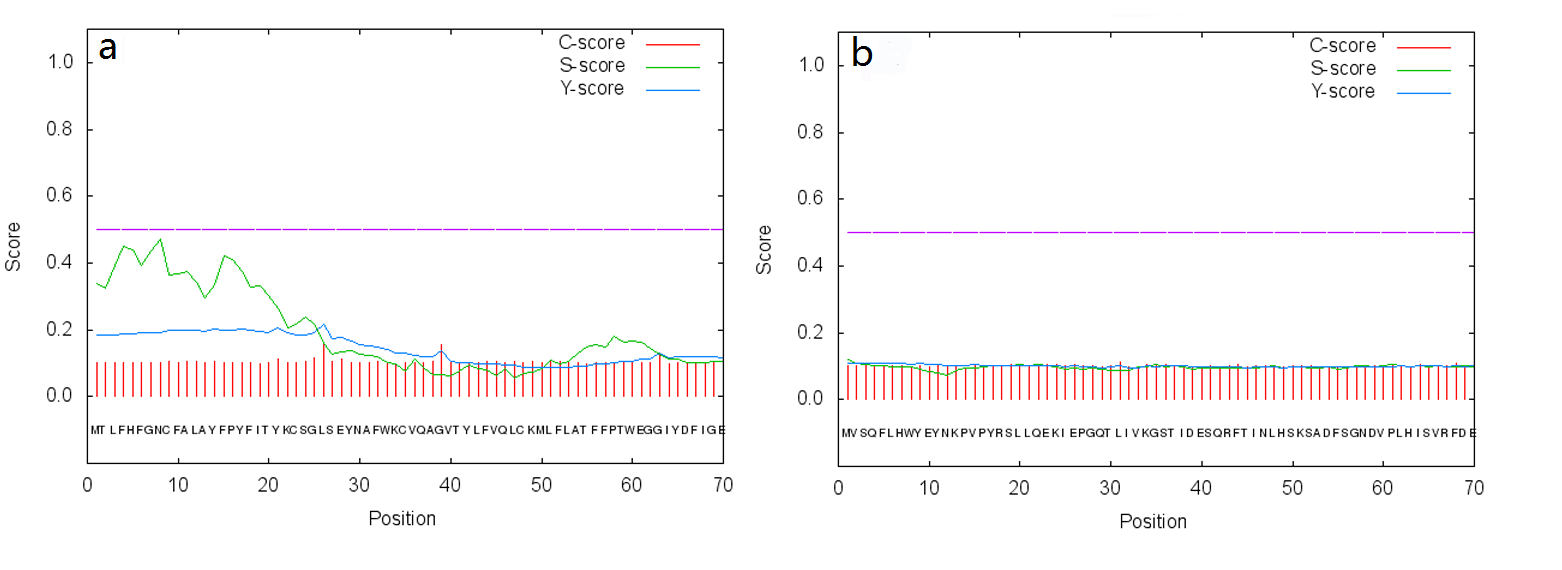


**Figure S2. Purification of recombinant TMEM147 and Hco-gal-m.** Purified recombinant proteins were resolved on 15 % acrylamide gels (a and b), and stained with coomassie brilliant blue R250. a, Recombinant ‘TMEM147-outside’ protein was approximately 12.13 kDa (including 7 kDa fusion proteins and a 5.13 kDa TMEM147-outside region); b, Recombinant Hco-gal-m protein was approximately 39.50 kDa (including 7 kDa fusion proteins and 32.50 kDa Hco-gal-m).


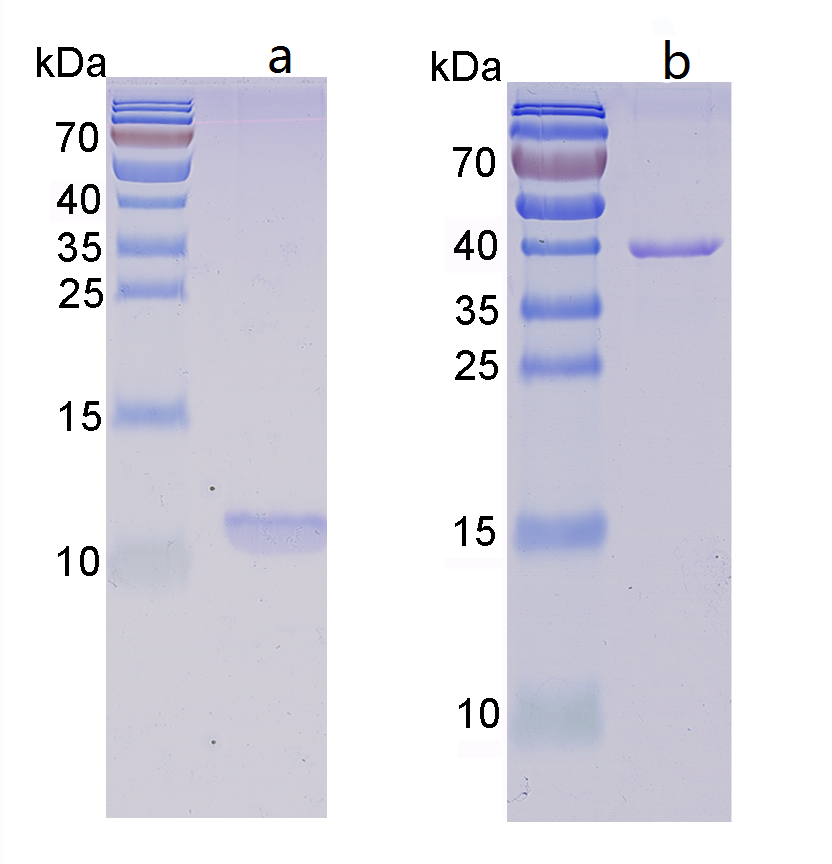


**Figure S3. Confirmation of polyclonal antibody specificity by western blot.** Goat PBMC were lysed with lysate buffer and loaded in gels in SDS loading buffer. The cell lysates (a and b) or recombinant Hco-gal-m without fusion proteins (c) were resolved on 12 % acrylamide gels (a, b and c). Proteins on gels were transferred onto 0.22 μm PVDF transfer membranes and were probed by incubating with anti-TMEM147-O IgG (a) or anti-Hco-gal IgG (b and c) primary antibodies. a, TMEM147 can be recognized by anti-TMEM147-O IgG, and the band was approximately 25.32 kDa; b, No band was observed in the cell lysates stained by anti-Hco-gal IgG; c, Recombinant Hco-gal-m without fusion proteins (the 4.91 kDa fusion proteins were cleaved by thrombin) could be recognized by anti-Hco-gal IgG and was approximately 34.59 kDa.


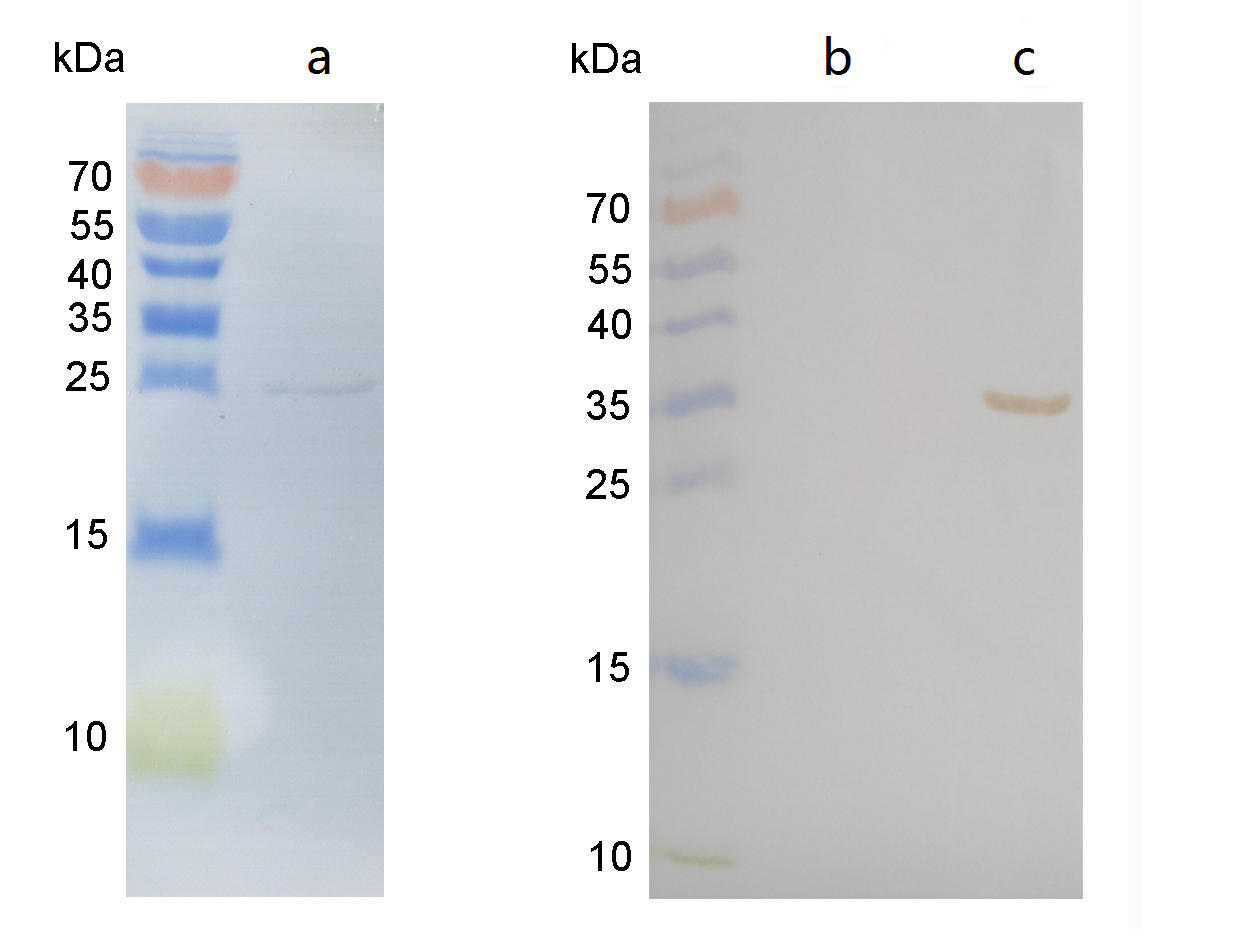


**Figure S4. Membrane protein prediction using TMHMM Server v.2.0.** The amino acid sequences of TMEM147 (NCBI accession numbers: JQ923484) were analyzed to predict transmembrane structures using TMHMM Server v.2.0. The proteins were predicted to contain 7 transmembrane domains.


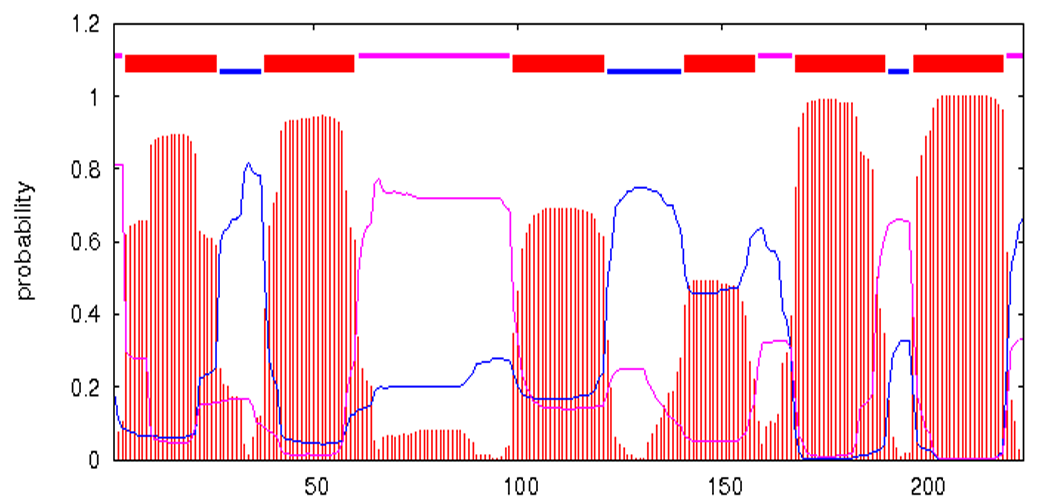


**Figure S5. The knockdown efficiency of TMEM147 at different time points.** Goat PBMC were transfected with TMEM147 siRNA. The level of TMEM147 mRNA transcript was reduced at 24 h (a) after RNAi treatment. An asterisk indicates that the value was significantly different (*P* < 0.05) from that of the 0 h group. A significant decrease in TMEM147 protein level was observed by western blotting at 48 h after RNAi (b, TMEM147). Lane 1 to 4 were loaded with cell lysates (10 μg/lane) harvested at 0, 24, 48 and 60 h after RNAi. Beta-actin was used as a protein-loading control (b, Beta-actin). The results presented here are from one independent experiment and are representative of three independent experiments.


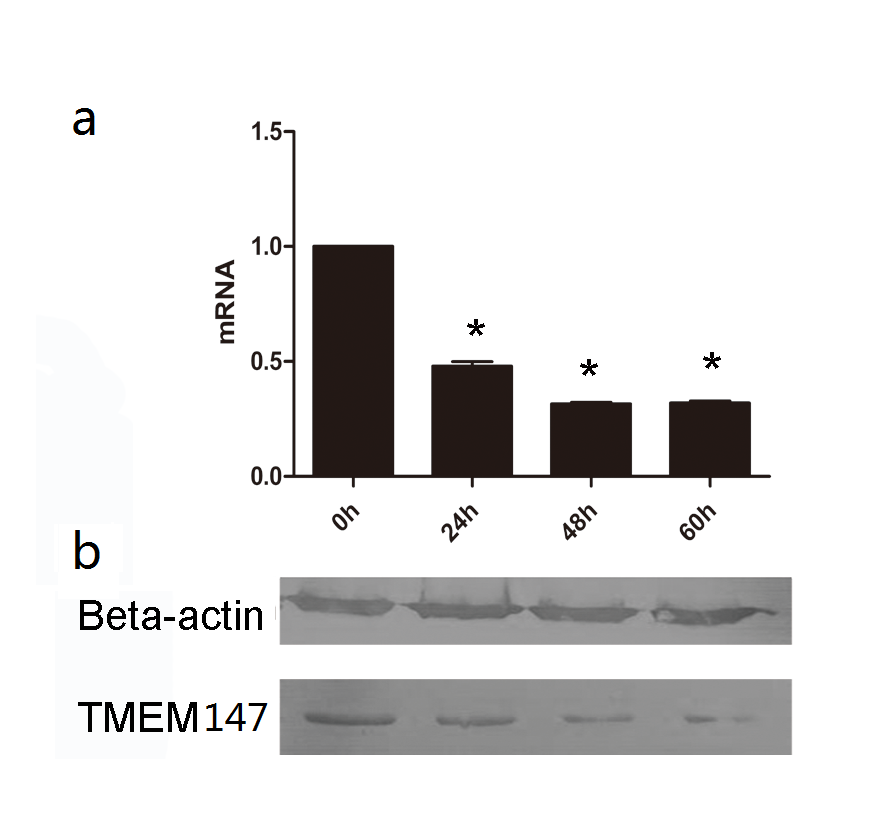

Supplement: Additional file 3: Figure S1. — N-terminal signal peptide prediction. Figure S2. Purification of recombinant TMEM147 and Hco-gal-m. Figure S3. Confirmation of polyclonal antibody specificity by western blot. Figure S4. Membrane protein prediction using TMHMM Server v.2.0. Figure S5. The knockdown efficiency of TMEM147 at different time points. (DOCX 1555 kb) [file 13071_2016_1640_MOESM3_ESM.docx]
